# Synthesis of Pyrodextrins and Enzymatically Resistant Maltodextrins from Makal (*Xanthosoma yucatenensis*) Starch

**DOI:** 10.17113/ftb.62.01.24.8163

**Published:** 2024-03

**Authors:** Enrique Barbosa-Martín, Enrique Sauri-Duch, Luis Chel-Guerrero, Luis Cuevas-Glory, Víctor Moo-Huchin, David Betancur-Ancona

**Affiliations:** 1 Department of Chemical and Biochemical Engineering, Instrumental Analysis Laboratory, National Technological Institute of Mexico, km 5 Mérida-Progreso Highway 97118 Mérida, Mexico; 2 Department of Food Science, Faculty of Chemical Engineering, Autonomous University of Yucatán, Peripheral North km. 33.5, Cadastral Table 13615, 97203 Mérida, Mexico

**Keywords:** resistant starch, dietary fiber, pyrodextrins, enzymatically resistant maltodextrins, makal, *Xanthosoma yucatenensis*

## Abstract

**Research background:**

Enzymatically resistant maltodextrins (ERM) are a resistant starch type 4, synthesized from native starch. They are obtained by the sequential application of two processes: pyrodextrinization, which produces pyrodextrins, and enzymatic hydrolysis, which produces ERM. In these processes atypical bonds are formed that confer pyrodextrins and ERM similar properties to dietary fiber, such as resistance to digestion. The aim of this work is to determine and evaluate some physicochemical properties of pyrodextrins and ERM obtained from native starch isolated from makal (*Xanthosoma yucatanense*) tubers.

**Experimental approach:**

Pyrodextrinization and complementary hydrolysis were conducted using factorial designs. For pyrodextrinization, factors and their levels were (*m*(starch):*V*(HCl))=80:1 and 160:1 (*c*(HCl)=2.2 M), temperature 90 and 110 °C and reaction time 1 and 3 h, and for CH, α-amylase per pyrodextrin volume fractions 0.5 and 1 µL/mL and reaction time 10 and 30 min. The physicochemical profile included determination of resistant starch content, estimation of color change (Δ*E*), microscopy and determination of dextrose equivalents (DE).

**Results and conclusions:**

According to the factorial design, the best treatment conditions for pyrodextrinization were: (*m*(starch):*V*(HCl))=160:1, 90 °C and 3 h, since they resulted in the highest resistant starch content (84.73 %) and the lowest Δ*E* (3.742). Due to the low DE (13.89 %), increased amount of resistant starch (90.73 %) and low Δ*E* (4.24) in the resulting ERM, complementary hydrolysis with α-amylase per pyrodextrin volume fraction 0.5 µL/mL and hydrolysis time 10 min was selected as the best treatment.

**Novelty and scientific contribution:**

The results show that the pyrodextrins and ERM obtained from makal can be used as ingredients for the development of functional foods, due to their high content of indigestible material and low degree of browning.

## INTRODUCTION

Starch is the main carbohydrate reserve in plants and the main source of energy in human and animal diets. It is also widely used in the food industry, either as a main raw material or as a food additive. Regarding their digestibility, according to the Englyst and Hudson (*1*), there are two types of starch whose characteristics are related to their physiological properties: digestible starch and resistant starch.

Digestible starch is defined as being susceptible to hydrolysis and absorption by the human small intestine (*2*). It consists of amylose and amylopectin chains, whose hydrolysis is mainly mediated by amylases, limit dextrinase and disaccharides. These enzymes hydrolyze the starch into glucose monomers that are rapidly absorbed, leading to an increase in postprandial blood glucose (*2*).

Resistant starch cannot be hydrolyzed *in vivo* by human digestive enzymes but can be fermented by the microbiota (*3*). At laboratory level, resistant starch cannot be hydrolyzed by incubation with α-amylase unless it is adequately dispersed with potassium hydroxide or dimethyl sulfoxide. Due to its antidiabetic, prebiotic and hypolipidaemic properties, among others, it can be considered a functional ingredient that increases food quality and could have a positive impact on consumer health (*3*).

Five types of resistant starch (RS) have been defined in scientific literature. Type 1 (RS1) is physically inaccessible to enzymes as it is protected and compacted by the cell wall in some grains and tubers. Type 2 (RS2), which is called "crude" and has a crystalline structure that cannot be assimilated, is found in raw potatoes and green bananas. The rest of resistant starches are obtained from various chemical-technological processes, by retrogradation after cooking (RS3), by chemical and/or enzymatic induction (RS4) and by conjugation with free fatty acids (RS5) (*1*, *3*).

Resistant starch type 4 in particular is formed by various chemical modifications such as repolymerization, crosslinking and substitution. All changes that starch can undergo increase indigestibility through specific mechanisms caused by the chemical reactions (*4*). Repolymerization (*5*) and cross-linking (*6*) increase atypical bonds which make starch indigestible. Chemical substitutions increase enzymatic resistance, probably due to bulky derivatization groups that sterically hinder the formation of enzyme-substrate complexes (*7*).

Enzymatically resistant maltodextrins (ERM) are classified within type 4 RS since their synthesis is induced by heat treatments, acidic conditions and enzymatic hydrolysis that form atypical bonds that are resistant to hydrolysis by digestive enzymes. ERMs are synthesized from native starch by the sequential application of two main processes: pyrodextrinization and complementary enzymatic hydrolysis (*8*).

In the pyrodextrinization or pyroconversion process, native starch is exposed to high temperatures over a long period of time using acid as a catalyst. It is performed under low humidity conditions and involves hydrolysis, transglycosylation and repolymerization reactions that promote the formation of atypical bonds, such as α-1-2, β-1-2, β-1-4, and β-1-6, which makes the substrate indigestible (*9*). Once the pyrodextrinization process is complete, the resulting dextrin-rich solution is hydrolyzed with amylases to remove the remaining bonds that make starch digestible (*10*). In this regard, Laurentin *et al.* (*11*) found that the digestible bonds in the available starch decrease by 55–65 % after pyroconversion. The product obtained from pyrodextrinization is then filtered, decolorized, deionized, concentrated and spray-dried. The resulting product, ERM, is characterized by its resistance to any hydrolysis by heat, acids or enzymes, including those of the digestive tract. Therefore, most ERMs can reach the large intestine almost intact, where they are fermented by microbiota (*10*).

Based on the abundant evidence in scientific literature on beneficial health effects of ERM such as indigestibility (*12*), fermentability and mass control (*13*), the present research aims to synthesize ERM from a non-conventional plant source of starch, makal (*Xanthosoma yucatenense*), by applying a factorial design based on response surface methodology. First, a pyrodextrinization process was developed (using three variables: *m*(starch)/*V*(acid) ratio, temperature and reaction time), followed by a complementary hydrolysis (using two variables: α-amylase per pyrodextrin volume fraction and reaction time) to finally synthesize ERM. It is important to mention that makal is a rhizome endemic to Mexico, specifically to the Yucatán Peninsula. It is rich in native starch with chemical, structural and nutritional properties similar to those of maize, potato, cassava, sago and sweet potato starch.

## MATERIALS AND METHODS

### Native starch extraction

Fresh makal obtained from the local market of the municipality of Kimbilá, Yucatán, Mexico (longitude 20°17'45" and latitude -89°05'05") was used. Makal tubers were harvested during the months of July and September 2022. The rhizomes were cleaned and stored in refrigeration at 4 ºC until use. The tubers were processed based on the method by Estrada *et al.* (*14*), with some modifications. Rhizomes were peeled, cut into 3-cm cubes and soaked for 30 min in a sodium hydrogen sulfite solution (1.5 g/L; (*m*(sample)/*V*(solution))=1:3). Cubes were then ground in a blender (Oster 465-42; Sunbeam Products Inc., Delray Beach, FL, USA) and the resulting mass was suspended in a sodium hydrogen sulfite solution (1.5 g/L; *φ*(sample,solution)=0.5). To remove mucilaginous material, the mixture was centrifuged twice at 1100×*g* for 12 min (Mistral 3000i centrifuge; SpectraLab Scientific Inc., Markham, ON, Canada) and the supernatant was discarded. The precipitate was suspended again with sodium hydrogen sulfite and filtered through 80 mesh (177 μm) in laboratory sieves (Cole-Parmer, Vernon Hills, IL, USA) to remove fiber. The filtrate was left to settle at 4 °C for 24 h. When phase separation was observed, the supernatant was removed by siphoning and the precipitate was washed with distilled water and centrifuged at 1100×*g* for 12 min (Mistral 3000i; SpectraLab Scientific Inc.). The obtained starch was dried in a convection oven (Thermo Fisher Scientific Inc., Waltham, MA, USA) at 55 °C for 24 h, milled in a laboratory mill (Ciclotec; Tecator, Stockholm, Sweden) to obtain a powder that could pass through 100 mesh (149 µm) and finally stored until further use.

### Pyroconversion

Native starch was chemically modified by pyroconversion process described by Toraya-Avilés *et al.* (*8*), with some modifications, taking into account the response surface methodology. A 2^3^ factorial design with four replicates of the central treatment was applied to evaluate the fit to a first-order polynomial model. The evaluated factors and levels were: (*m*(starch):*V*(HCl))=80:1 and 160:1 (*c*(HCl)=2.2 M), temperature 90 and 110 °C and reaction time 1 and 3 h. A mass of 15 g of native starch (dry basis) was placed in a Petri dish and 1 mL of 2.2 M HCl was added. The acid was sprayed into the starch and allowed to react in a desiccator for 16 h. The product was then dried in a convection oven (Thermo Fisher Scientific Inc.) at the appropriate temperature and time. When statistical analysis showed a significant effect (p<0.05) due to the lack of the first-order model fit, additional experiments were performed to optimize the pyroconversion. Six axial points were included (α=[2^n^]^1/4^, where n=number of independent variables, in this case: [2^3^]^1/4^ =1.683) for the optimization of a second-order model.

The pyrodextrins obtained from each treatment were milled, sieved through an 80 mesh (177 µm) in laboratory sieves (Cole-Parmer) and stored in a desiccator. To select the best treatment, RS content was used as the only response variable, with color difference (Δ*E*) relative to native starch as a complement. The best pyrodextrin was determined to be the one where the RS was higher and the Δ*E* lower. In the food industry, colorless dextrin in a solution is desirable. New dextrins are best when they are colorless, as any coloration resulting from pyroconversion limits their uses, and eliminating the need for color removal reduces costs.

Indigestible material was quantified according to the guidelines described in Megazyme® Resistant Starch Kit Cat. No. K-RSTAR 05-19 (Megazyme International, Brey, Ireland) (*5*). This method consists of a hydrolysis using amyloglucosidase and α-amylase, followed by a solubilization process with ethanol. Finally, a dispersion with KOH is performed and RS is quantified by interpolation with the absorbance (*A*) of a standard glucose solution (1 mg/mL) using the following equation:


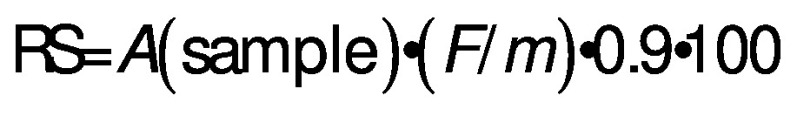
 /1/

where *A* is the absorbance of the sample at 510 nm, *F* is *c*(standard)/*A*(standard), *m* is dry mass of sample in g and 0.9 is the conversion factor of starch to glucose.

Both color and Δ*E* were determined based on the Hunter scale (*15*). Using a Gardner 6805 photoelectric colorimeter (BYK-Gardner GmbH, Geretsried, Germany), color was identified in three attributes: *L* (white=100, black=0), *a* (red=positive, green=negative) and *b* (yellow=positive, blue=negative). Δ*E* was estimated using the color of native starch as a standard, using the following equation:


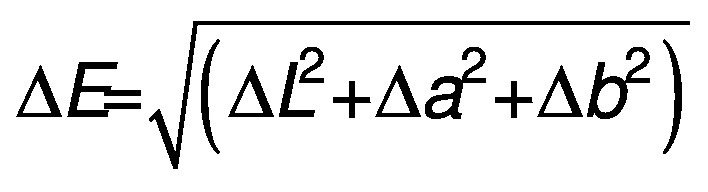
 /2/

where Δ*E* is color change, Δ*L* is color change in *L* attribute, Δ*a* is color change in *a* attribute and Δ*b* is color change in *b* attribute.

Changes in granular structure were evaluated by microscopic observations of native starch and pyrodextrin obtained under the best treatment conditions. For this purpose, a 0.5 g/100 mL solution of starch or pyrodextrin was prepared with distilled water and observed under an optical microscope (OMAX 14000 KPA; Kitchener, Ontario, Canada) at 40× magnification. The images were processed with OMAX Toup View 3.7 software (OMAX 14000 KPA, Kitchener) according to the method of Zeng *et al*. (*16*).

### Complementary enzymatic hydrolysis

Once the best pyrodextrin was selected, α-amylase (Sigma-Aldrich, Merck, St. Louis, MO, USA) was used for hydrolysis according to the procedure described by Toraya-Avilés *et al.* (*12*) to synthesize ERM. A 2^2^ factorial design with four replicates of the central treatment was used. The factors and their levels were: *φ*(enzyme,substrate)=0.5 and 1 µL/mL and reaction time 10 and 30 min. Pyrodextrin was placed in a water suspension (0.2 g/mL) and the pH was adjusted to 6 with 1 M NaOH. It was then shaken, incubated at 95 °C in a bath (R76; New Brunswick Scientific Co Inc., Edison, NJ, USA) and the enzyme was added at respective volume fractions (0.05 or 0.1 mL per 100 mL of starch suspension) for the reaction times mentioned above. After completion of hydrolysis, the enzyme was inactivated at 120 °C for 15 min in a convection oven (Thermo Fisher Scientific Inc.). Finally, the obtained sample was spray-dried, milled and sieved through an 80 mesh (177 µm). A spray dryer SD-05 Lab Plant (Keison Products, Essex, UK) with an inlet temperature of 135 °C and outlet temperature of 85 °C, a feed nozzle diameter of 0.75 mm and a drying air flow and a sample feed pump capacity of 62 m^3^/h and 560 mL/h, respectively, was used for spray drying.

Number of dextrose equivalents (DE) was used as a response variable to determine the best treatment. DE is defined as the percentage of reducing sugars from a hydrolyzed polysaccharide, calculated as glucose on a dry mass basis. Therefore, the DE of starch is zero and that of glucose is 100. A useful application of this parameter is in determining maltodextrins because they must not have more than 20 % reducing sugars (*i.e.*DE<20) to be defined as maltodextrins (*17*). DE were quantified in all complementary hydrolysis treatments to select the best ERM. Based on the method described by Miller (*18*), 1 g of sample was weighed and adjusted to 100 mL with distilled water. From this solution 1 mL was taken, 1.5 mL of 3,5-dinitrosalicylic acid was added and heated to boiling for 15 min. Then, the sample was read in a Cary 60 UV-Vis spectrophotometer (Agilent Technologies, Santa Clara, CA, USA) at 550 nm. The content of reducing sugars was quantified by interpolation of absorbance values obtained from samples related to the glucose standard curve (0, 0.2, 0.4, 0.6, 0.8 and 1.0 mg/mL). The result was expressed as a percentage based on total starch used and the best ERM was determined as the one with a DE value <20 (equivalent to <20 % reducing sugars), based on statistical analysis. Once the best ERM was selected, its resistant starch content and Δ*E* were determined according to the previously described methods.

### Statistical analysis

The obtained results were analyzed by analysis of variance with a 95 % confidence level. Duncan's multiple range test was applied to determine the differences between treatments. Regression analysis was performed using StatGraphics Centurion software v. 18 (*19*) according to the method reported by Montgomery (*20*).

## RESULTS AND DISCUSSION

### Pyroconversion: First order modeling

As a result of the factorial experiment for pyrodextrin synthesis, a formation of 46.19–84.73 % of resistant starch (RS) was observed compared to 5.69 % native starch. Treatment 5 was found to be the best for pyrodextrin production (p<0.05). Experimental conditions for this treatment were: (*m*(starch)/*V*(acid))=160:1, temperature 90 °C and reaction time 180 min (Table 1). RS as dependent variable showed that a first-order model was followed in relation to independent variables, and coefficient of determination (R^2^) value revealed that the model explains 87.43 % of the observed data. The formation of RS in pyrodextrins in relation to the factors is described by the following equation:


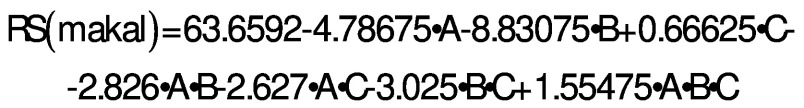
 /3/

**Table 1:** Effect of starch and HCl ratio, temperature and time on the formation of pyrodextrins derived from makal starch (first-order experiment)

Treatment	Factors and levels (natural and codified)			
	m(starch)/V(acid)	Temperature/°C	Time/min	Resistant starch/%
1 (Factorial)	160:1 (-1)	90 (-1)	60 (-1)	68.99^h^
2 (Factorial)	80:1 (+1)	90 (-1)	60 (-1)	73.43^i^
3 (Factorial)	160:1 (-1)	110 (+1)	60 (-1)	66.14^g^
4 (Factorial)	80:1 (+1)	110 (+1)	60 (-1)	53.06^c^
5 (Factorial)	160:1 (-1)	90 (-1)	180 (+1)	84.73^j^
6 (Factorial)	80:1 (+1)	90 (-1)	180 (+1)	72.45^i^
7 (Factorial)	160:1 (-1)	110 (+1)	180 (+1)	63.56^f^
8 (Factorial)	80:1 (+1)	110 (+1)	180 (+1)	46.19^b^
9 (Central)	120:1 (0)	100 (0)	120 (0)	59.95^e^
10 (Central)	120:1 (0)	100 (0)	120 (0)	59.86^e^
11 (Central)	120:1 (0)	100 (0)	120 (0)	58.83^d,e^
12 (Central)	120:1 (0)	100 (0)	120 (0)	56.67^d^
Native	-	-	-	5.69^a^

Different letters in superscript in the same column indicate significant difference (p<0.05). *c*(HCl)=2.2 M

where A is *m*(starch)/*V*(acid), B is temperature and C is time.

Analysis of variance revealed that time (C) and the triple interaction of factors starch/acid ratio×temperature×time (ABC) did not affect RS yield significantly (p>0.05). On the other hand, starch/acid ratio (A), temperature (B) and two-factor interactions (AB, AC and BC) had a statistically significant (p<0.05) effect on RS formation. Considering the coefficients of regression equation (Eq. 3), it can be inferred that these same significant factors negatively affect yield.

These results are in agreement with most reports on the effect of acid and hydrolysis time on pyrodextrins. Similar to the observations in the present work, Toraya-Avilés *et al.* (*8*) found that factors that negatively affected RS yield in cassava pyrodextrins were starch/acid ratio, temperature and temperature×time. However, their RS yield was ~50 % lower than the one obtained in this study. A practical interpretation of the effects of the factors must consider the numerical value of the regression coefficients and the plus or minus sign of these coefficients. The larger the coefficient, the greater the impact of the factor or independent variable on the response variable, in this case, the RS content. A coefficient with the plus sign indicates that the factor (starch/acid ratio, temperature, and temperature×time) positively affects the RS content; to promote its formation, high levels of the variable should be used. Similarly, a regression coefficient with a minus sign indicates that low levels of the factor should be used to increase the RS content during the reaction. Chen *et al.* (*21*) explained this negative effect of acid and temperature on the maize pyrodextrins by the fact that catalytic acid is an important promoter of starch hydrolysis. Therefore, acid hydrolysis leads to a reduction in RS formation and an increase in digestibility. According to these authors, this scenario can be reproduced with the treatments at high temperature. Lin *et al.* (*22*) also stated that the combination of acid and temperature under extreme conditions leads to the partial decomposition of starch into gaseous compounds and consequently a decrease in pyrodextrin yield.

As the lack of fit of the model was statistically significant (p<0.05), it became clear that the first-order model was not sufficient to describe the observed data. This required further experiments to move to a higher order model to explain RS formation.

### Pyroconversion: Second order modeling

After the application of axial treatments, a range of RS between 46.19 and 84.7 % was maintained. A significantly higher percentage of RS (84.73 %) was observed in treatment 5 with a (*m*(starch)/*V*(acid))=160:1, temperature 90 °C and reaction time 180 min (Table 2). Analysis of variance revealed that factors and interactions that did not significantly (p>0.05) affect RS yield were C, AA and ABC. Factors A and B, as well as the double interactions AB, AC, BB, BC and CC had a significant effect (p<0.05), while BB and CC were the only ones that positively affected RS formation. The following equation describes the behavior of RS formation in relation to the evaluated factors:


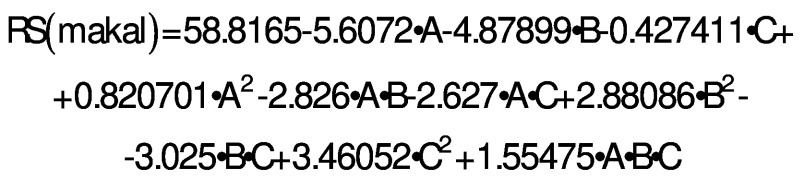
 /4/

**Table 2:** Effect of starch and HCl ratio, temperature and reaction time on the formation of pyrodextrins from makal starch (second-order experiment)

Treatment	Factors and levels (natural and codified)				
	*m*(starch)/*V*(acid)	Temperature/°C	Time/min	Resistant starch/%	Color change (Δ*E*)
1 (Factorial)	160:1 (-1)	90 (-1)	60 (-1)	68.99^h,i,j^	2.24^c^
2 (Factorial)	80:1 (+1)	90 (-1)	60 (-1)	73.43^k^	3.33^d^
3 (Factorial)	160:1 (-1)	110 (+1)	60 (-1)	66.14^g,h^	10.51^h^
4 (Factorial)	80:1 (+1)	110 (+1)	60 (-1)	53.06^c,d^	13.99^j^
5 (Factorial)	160:1 (-1)	90 (-1)	180 (+1)	84.73^l^	3.74^d^
6 (Factorial)	80:1 (+1)	90 (-1)	180 (+1)	72.45^j,k^	5.14^f^
7 (Factorial)	160:1 (-1)	110 (+1)	180 (+1)	63.56^f,g^	12.84^i^
8 (Factorial)	80:1 (+1)	110 (+1)	180 (+1)	46.19^b^	20.13^k^
9 (Central)	120:1 (0)	100 (0)	120 (0)	59.95^e,f^	8.18^g^
10 (Central)	120:1 (0)	100 (0)	120 (0)	59.86^e,f^	8.24^g^
11 (Central)	120:1 (0)	100 (0)	120 (0)	58.83^e^	8.41^g^
12 (Central)	120:1 (0)	100 (0)	120 (0)	56.67^d,e^	8.25^g^
13 (α, axial)	53:1 (1.68179)	100 (0)	120 (0)	49.62^b,c^	8.51^g^
14 (α, axial)	187:1 (-1.68179)	100 (0)	120 (0)	72.38^j,k^	3.16^d^
15 (α, axial)	120:1 (0)	117 (1.68179)	120 (0)	68.02^h,i^	10.55^h^
16 (α, axial)	120:1 (0)	83 (-1.68179)	120 (0)	65.63^g,h^	2.24^c^
17 (α, axial)	120:1 (0)	100 (0)	221 (1.68179)	65.15^g,h^	4.46^e^
18 (α, axial)	120:1 (0)	100 (0)	19 (-1.68179)	71.79^i,j,k^	1.90^b^
Native				5.69^a^	0^a^

Axial points: α=(2^n^)^¼^, n=number of independent variables. In this case: (2^3^)^¼^=1.683. Different letters in superscript in the same column indicate significant difference (p<0.05). *c*(HCl)=2.2 M

where A is *m*(starch)/*V*(acid), B is temperature and C is time.

When comparing indigestible content of different pyrodextrins reported in the literature, the value of 84.73 % obtained in the present study (treatment 5; Table 2) clearly stands out. As mentioned above, this could be due to the extreme conditions of temperature, acidity and time. The negative effect of temperature may be reflected in the pyrodextrins obtained from rice starch, as it was shown that a low percentage of RS (22.26–27.14 %) was obtained at 130° C for 1−3 h (*23*). In the work of Lin *et al*. (*22*) in addition to the effects of temperature, it was also observed that acid concentration negatively affects the final RS content. These authors pyrodextrinized maize starch at very high temperatures (140–180 °C) with high amounts of acid (0.5–2.5 %) and obtained an initial yield of indigestible material of 12.4 %.

In addition, it seems that even the use of mild conditions during pyroconversion negatively affects RS yield. In pyrodextrinized potato starch, a content of 14.5–21.5 % of indigestible material was reported by Kapusniak *et al.* (*24*), who used a lower amount of acid (0.1 %) and less time (2 h) but a similar temperature (110 °C) to the one used in the present study. Jochym and Nebesny (*25*) reported a similar content of indigestible material (∼25 %) in pyrodextrinized potato starch under similar conditions. Despite the low yield, these authors described their resistant pyrodextrins as a potential ingredient in functional beverages due to their high solubility and low viscosity.

In their work with various starches (cassava, malanga, lentil, maize, sago and sorghum), Laurentin *et al*. (*11*) found that the use of acid catalysts and temperature is essential for effective pyroconversion processes. According to these authors, dextrinization with acid and moderate temperature is ideal for generating dehydration and intramolecular rearrangement, which favors the formation of atypical indigestible bonds.

RS yield obtained in the present study can be explained by various structural changes in the native starch. Le Thanh *et al.* (*26*) found that the transglycosylation that occurs during pyrodextrinization is responsible for the replacement of the α-1,4 bonds that make starch digestible by atypical bonds such as α-1,2, α-1,3, β-1,4 and β-1,6. In a chemical structure analysis using nuclear magnetic resonance (NMR) spectroscopy, it was shown that, in addition to transglycosylation, other phenomena associated with the formation of bonds making starch indigestible are depolymerization and branching. In particular, this study showed that the total number of α-1,4 bonds increased by 12 % after pyrodextrinization of maize starch (*27*). The formation of steric hindrance in the enzyme action site (*8*) and retrogradation (*28*) are structural changes associated with increased indigestibility as a result of pyrodextrinization.

The determination coefficient (R^2^) for the mathematical model to explain RS formation was 77.42 %. Since the p-value for the lack of fit test was 0.007, this indicated that the second order model was not sufficient to represent the data and that the experiment could therefore be continued with a higher order model. However, due to the high content of indigestible material (84.73 % in treatment 5), the technological implications and the cost-benefit ratio of further experiments, it was decided not to extend the pyroconversion to a higher order model.

### Color change and microscopy

As a complementary indicator for the selection of the best pyroconversion treatment, Δ*E* was considered. The pyrodextrin in treatment 5, which had the highest RS content, had a slight color change (Δ*E*=3.74) compared to the control (native starch) (Table 2). The Δ*E* of pyrodextrin in treatment 5 was lower (p<0.05) than most treatments and only equal (p>0.05) to treatments 2 and 14, whose experimental conditions were: (*m*(starch)/*V*(acid))=80:1 and 187:1, temperature 90 and 100 °C and time 60 and 120 min, respectively (Table 2).

It is worth noting that the Δ*E* observed in pyrodextrins is the result of browning due to starch oxidation, which leads to the formation of carbon and/or low-molecular-mass carbonyl compounds (*21*). This browning reaction is highly dependent on the pyroconversion conditions, especially temperature and acidity (*28*). This is consistent with the results of the present study, as the analysis of variance indicated that all factors had significant effects (p<0.05) on Δ*E*.

Regarding temperature, Toraya-Avilés *et al.* (*8*) obtained a low Δ*E* (1.89) in their optimal pyrodextrinization treatment with a moderate temperature, similar to that of the present study (90 °C). In contrast, Weil *et al.* (*29*) show the effects of a treatment with high temperatures on pyrodextrin browning. These authors obtained almost three times higher Δ*E* in their pyrodextrin synthesized at almost twice the temperature (170 °C) and a longer reaction time (4 h) than in the present study (9.2–10.6). Regarding the catalytic acid, Lin *et al.* (*22*) found that the degree of color change of pyrodextrins depends on the type and concentration of acid, with the Δ*E* being more pronounced when HCl was used. These authors obtained highly browned pyrodextrins when using HCl (Δ*E*=70.5), compared to glacial acetic acid (Δ*E*=34.2).

**Fig. 1:**
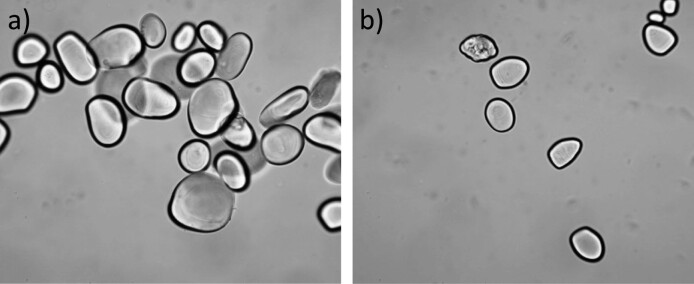
Micrographs of: a) native starch and b) pyrodextrin from the best treatment ((*m*(starch)/*V*(acid))=160:1, temperature=90 °C, reaction time=180 min)

To confirm the effect of pyroconversion, the pyrodextrin from treatment 5 (considered the best due to its higher RS content (84.7 %) and low Δ*E* (3.74)) was observed under the microscope (Table 2). This shows that the acid-thermal treatment hydrolyzed a large part of granules of makal native starch (Fig. 1). This was an expected phenomenon since an implicit process during pyroconversion reactions is structural-spatial degradation of starch granules (*26*, *28*, *30*).

### Complementary enzymatic hydrolysis: Obtaining ERM

The pyrodextrin from treatment 5 was treated with α-amylase to hydrolyze bonds in digestible part of starch remaining after pyroconversion, to increase the proportion of atypical bonds and promote enzymatic resistance. The hydrolyzed carbohydrate residue was used as an indicator to estimate dextrose equivalents (DE) and identify the sample as maltodextrin. Therefore, the sample that had the highest amount of RS and the lowest DE (<20 %) was defined as the best enzymatically resistant maltodextrin (ERM).

The analysis of variance showed that there were statistical differences among the treatments (p<0.05). Duncan's multiple range test showed that treatments 2, 4 and the central points (5-8) were statistically equal (p<0.05). Factorial treatment 1 was selected because it had the statistically lowest DE value (Table 3). It was determined that the two analysed factors had significant effects (p<0.05) on DE.

**Table 3:** Dextrose equivalents in enzymatically resistant maltodextrins from makal starch

Treatment	Factors and levels		
	(*V*(enzyme)/*V*(substrate))/(µL/mL)	Reaction time/min	Dextrose equivalent/%
1 (Factorial)	0.5	10	13.89^a^
2 (Factorial)	1.0	10	36.56^b^
3 (Factorial)	0.5	30	24.24^c^
4 (Factorial)	1.0	30	33.85^b^
5 (Central)	0.75	20	36.18^b^
6 (Central)	0.75	20	35.70^b^
7 (Central)	0.75	20	38.03^b^
8 (Central)	0.75	20	32.29^b^

Different letters in superscript in the same column indicate significant difference (p<0.05)

The degree of starch hydrolysis was expressed by the number of dextrose equivalents (DE) remaining after hydrolysis. Glucose DE was 100 and starch was zero. One application of this parameter is to define maltodextrins, as to be labelled as maltodextrin, they must not contain more than 20 % sugars and have a DE<20 (*31*). For the purpose of this study, DE was used to determine the best ERM for complementary hydrolysis. Since the ERM produced with 0.5 µL/mL enzyme in a 10-min hydrolysis (treatment 1) had a DE<20 and this value was statistically lower than those of the rest of the treatments (Table 3), it was selected as the most suitable for the synthesis of the makal starch derivative. RS content and its Δ*E* were determined again for this sample. It was found that the percentage of indigestible material increased to 90.73 % and the color change to 4.24.

As previously mentioned, the best ERM was obtained with the lowest levels of the evaluated factors (treatment 1; Table 3). These results are consistent with those reported in the literature, since it has been shown that low enzyme amounts with shorter enzyme-dextrin contact times negatively affect hydrolysis rates and produce lower DE values (*8*).

Considerable increase in RS to 90.7 % in the ERM was mainly due to the fact that complementary hydrolysis was a synergistic and sequential treatment to pyrodextrinization; a process in which 84.73 % of RS had already been produced (Table 2) as a result of the structural changes described above. Moreover, it has been demonstrated that complementary hydrolysis tends to induce a complex structural rearrangement leading to an increase in indigestible material (*25*). Astina and Sapwarobol (*10*) have detected that during hydrolysis new aldehyde ends are generated as a result of cleavage of remnant bonds α-1,4 and α-1,6, which tend to form hydrogen bonds with the -OH ends of glucose at random positions, thus forming atypical bonds (such as α-1,2 and α-1,3) and increasing the content of indigestible material.

In this study, an enzymatically resistant maltodextrin with 90.73 % RS was found, which is much higher than other reported ERMs. Toraya-Avilés *et al*. (*8*) and Trithavisup *et al.* (*32*) obtained RS amounts of 45.98 and 61.76 %, respectively, in their ERM obtained from cassava. Moreover, our RS yield is higher than other type 4 RS, such as those reported by Ashwar *et al.* (*33*) with 45.35 % and Xia *et al.* (*34*) with 70.57 %.

The percentage of indigestible material in the ERM of the present study is similar to Nutriose® (85 %) (*35*) and Fibersol® (90 %) (*36*), both maltodextrin-based resistant products currently marketed for human consumption.

This high amount of indigestible components represents an enormous advantage. From a technological point of view, several studies have shown how pyrodextrins and ERMs could be used as food additives due to their wide range of physicochemical properties, such as low browning (*8*), low molecular mass (*26*), low viscosity, high solubility and a low tendency to retrogradation (2*7*). Its use was reported mainly in low-calorie foods (*10*) with favorable sensory attributes (*37*). Foods with this type of fiber include beverages, confectionery, extruded dehulled adlay (*38*), bakery products (*39*), and milkshakes (*40*), among others.

ERM in particular has been of great physiological interest as it behaves as soluble dietary fiber due to its high indigestibility (not assimilable) and solubility (*12*). The high hydration capacity of ERM guarantees the formation of hydrated reticular structures after consumption, which improve chyme transit in the intestine and bind compounds such as glucose (*10*, *40*). Since approx. 90 % of ERMs reach the large intestine and 50 % are fermented (*41*), they have been shown to function as prebiotics and can increase the production of short-chain fatty acids (*30*). In addition, there are reports that justify their use for satiety (*42*), mass control (*13*) and control of blood sugar levels (*40*). All this allows us to theorize that the ERM from makal obtained in this study could be associated with positive metabolic profiles when used as a functional food ingredient.

## CONCLUSIONS

Pyrodextrins and enzymatically resistant maltodextrins (ERM) were synthesized from makal (*Xanthosoma yucatanensis*) native starch. The best conditions for obtaining pyrodextrins were: (*m*(starch)/*V*(acid))=160:1, temperature 90 °C and reaction time 180 min. These conditions ensured a resistant starch (RS) content and a Δ*E* of 84.7 % and 3.74, respectively. Regarding ERM, the best treatment was obtained with 0.5 µL/mL α-amylase and a reaction time of 10 min. In this sample, RS content increased to 90.73 % and Δ*E* to 4.24.

The high proportion of indigestible material and low browning value, together with technological (low viscosity and low retrogradation, among others) and physiological (high solubility and high fermentability, among others) properties confirmed in other studies ensure that both pyrodextrins and ERMs can be used as functional ingredients that improve food quality.

It is therefore recommended to continue the studies on the extraction, characterization and biological evaluation of pyrodextrins and enzymatically resistant maltodextrins derived from makal starch in order to know all the mechanisms associated with their physicochemical properties and elucidate their related physiological effects.
